# Long-Term Depression in the Hippocampal CA1 Area of Aged Rats, Revisited: Contribution of Temporal Constraints Related to Slice Preparation

**DOI:** 10.1371/journal.pone.0009843

**Published:** 2010-03-24

**Authors:** Jean-marie Billard

**Affiliations:** Faculté de Médecine René Descartes, Centre de Psychiatrie et Neurosciences, Université Paris Descartes, UMR894, Paris, France; University of Alberta, Canada

## Abstract

**Background:**

The effects of low-frequency conditioning stimulation (LFS, 900 pulses at 1 Hz) of glutamatergic afferents in CA1 hippocampal area using slices from two different strains of adult (3–5 month-old) and aged (23–27 month-old) rats were reinvestigated regarding the discrepancies in the literature concerning the expression of long-term depression (LTD) in the aging brain.

**Methodology/Principal Findings:**

N-methyl-D-aspartate receptor (NMDA-R) dependent LTD was examined in both adult (n = 21) and aged (n = 22) Sprague-Dawley rats. While equivalent amounts of LTD could be obtained in both ages, there was significant variability depending upon the time between the slices were made and when they were tested. LTD was not apparent if slices were tested within 3 hours of dissection. The amount of LTD increased over the next three hours but more in adult than in aged rats. This age-related impairment was abolished by exogenous d-serine, thus reflecting the reduced activation of the NMDA-R glycine-binding site by the endogenous agonist in aged rats. Then, the amount of LTD reached asymptote at 5–7 hours following dissection. Similar temporal profiles of LTD expression were seen in young and aged Wistar rats.

**Conclusions/Significance:**

Taken together, these results sound a cautionary note regarding the existence of an experimental “window of opportunity” for studying the effects of aging on LTD expression in hippocampal slice preparation.

## Introduction

Activity-dependent changes in synaptic strength, such as long-term potentiation (LTP) and long-term depression (LTD) of synaptic transmission, are now considered leading candidates for the cellular mechanisms underlying learning and memory [1], [2], [3], [4], [5]. Competitive interactions between these specific forms of synaptic plasticity have been reported to underlie the storage of emotional memories and stress-induced amnesia [3], [6]. In fact, LTD may significantly determine the efficacy of learning and memory by limiting acquisition and favoring the decline of memory [7], [8], [9]. Because aging is generally associated with memory impairment, changes in susceptibility to LTD have been postulated to occur in the aged brain (see [10]). Although this question has been repeatedly investigated in the rat hippocampus, how LTD expression is affected by age still remains the subject of debate. On the one hand, an increase in susceptibility to LTD induction has been reported in aged animals [11], [12], [13], [14], [15], [16], [17] whereas on the other hand, several studies point more towards a weaker ability of hippocampal neuronal networks to generate synaptic depression with age [18], [19], [20]. Recent evidence also shows that strong stimuli do not induce any age-related changes in the magnitude of asymptotic LTD [21]. In their respective contributions, the authors have raised the possibility of inter-strain variability or methodological differences (such as different calcium/magnesium (Ca^2+^/Mg^2+^) ratio) to account for these contradictory results. However, it is not possible to confirm the relevance of these explanations since both increased and decreased susceptibility to LTD induction have been found in the same strain and/or for the same Ca^2+^/Mg^2+^ ratio (for a comparison of these studies see [15], [17], [18], [20].

In studying the effects of acute stress on metabotropic glutamate receptor (mGlu-R)-dependent LTD in hippocampal CA1 area, Chaouloff et al. have recently reported that application of the mGlu-R agonist (*RS*)-3,5-dihydrophenylglycine (DHPG) promotes LTD in slices from young animals subjected to a 4–7 h period of rest before recording whereas no effect was observed after a recovery period of 1–4 h period [22]. Although this point has not been discussed by the authors, it suggests that the ability of slices to express LTD is temporally related to rest conditions, possibly through basal intracellular free calcium [Ca^2+^]i concentrations, since a moderate increase in [Ca^2+^]i is a prerequisite for the induction of LTD [23], [24], [25], [26]. Interestingly, the increase in [Ca^2+^]i levels is dependent on the period for which the slices are maintained *in vitro* and the magnitude of this effect is significantly larger in slices from aged than from young ones [27]. In the present study, I therefore investigated age-related changes in susceptibility to LTD as a function of the resting time of the slices, in order to establish whether the discrepancies in the literature were a reflection of different conditions of the slice preparation.

## Methods

Experiments were carried out in accordance with the European Communities Council Directive (86/609/EEC) regarding the care and use of animals for experimental procedures and approved by the local ethical committee (Comité regional d'éthique en experimentation animale Paris Descartes, Université Paris 5). The experiments were conducted using adult (3–5 month-old, n = 21) and aged (23–27 month-old, n = 22) male Sprague-Dawley rats and adult (n = 8) and aged (n = 6) Wistar rats purchased from IFFA-CREDO.

One animal was studied per day. The rat was anesthetized with halothane and decapitated. The hippocampus was quickly removed and placed in ice-cold artificial cerebrospinal fluid (aCSF). The composition of the aCSF was as follows (in mM): NaCl 124, KCl 3.5, MgSO_4_ 1.5, CaCl_2_ 2.5, NaHCO_3_ 26.2, NaH_2_PO_4_ 1.2, and glucose 11, pH 7.4 (Ca^2+^/Mg^2+^ ratio = 1.6). This solution had a pH of 7.35 by bubbling a gas mixture of 95% O_2_/5% CO_2_. Its osmolarity was adjusted to 300 mOsm with an osmometer. In one series of experiments, the concentration of MgSO_4_ was raised to 2.5 mM so as to reach a Ca^2+^/Mg^2+^ ratio of 1. Slices (400 µm thick) were cut and placed into aCSF warmed at 28–30°C in a holding chamber to facilitate recovery for at least 1 hr. A single slice was then transferred to the recording chamber and continuously submerged with the pre-gassed aCSF.

Extracellular recordings were obtained at 25–28°C from the apical dendritic layer of CA1 area using glass micropipettes filled with 2 M NaCl (resistance 2–8 MΩ). Field excitatory postsynaptic potentials (fEPSPs) were evoked by electrical stimulation of afferent fibers (Schaffer collaterals and commissural fibers) located in the *stratum radiatum*. Stimuli (20 µs duration) were applied between the two poles of a bipolar tungsten electrode, with one pole inserted into the slice (350 µm diameter) and the other in the bath just above the slice.

Age-related effects on LTD were determined using test stimuli applied every 10 sec and adjusted to get a baseline slope of 0.2 mV/ms. After a period of at least 10 min for stabilization, the initial slope of three averaged fEPSPs was recorded and measured for 10 min using the Acquis 1 software (CNRS, Paris) before the delivery of a low-frequency conditioning stimulation (LFS, 900 pulses, 1 Hz at test intensity). Single pulses were then resumed for 40 min following LFS. The amplitude of the stimulus artifact was also continuously–monitored during the recording to ensure that changes in the fEPSP slope did not reflect alterations in tissue resistance.

Pharmacological experiments were performed in the presence of the antagonist D-2-amino-5-phosphonovalerate (d-APV, 80 µM) to block NMDA-R or the NMDA-R co-agonist d-serine at 100 µM that saturates NMDA-R glycine binding sites [28]. Drugs were added to the aCSF 10 min prior to baseline acquisition and maintained until the end of the experiment.

Data are reported as means ± SEM. Susceptibility to LTD expression was statistically estimated using analysis of variance (ANOVA) for repeated measures, carried out between the 10 min of baseline recordings and the last 10 min of recordings (i.e. 30 to 40 min after LFS). Age-related differences were determined using repeated measures ANOVA for the last 10 min of recordings. Post hoc analyses were performed using Scheffe's F-test with the threshold for significance set at *P*<0.05.

## Results

### Age-related susceptibility to LTD in control aCSF

**Figure 1:**
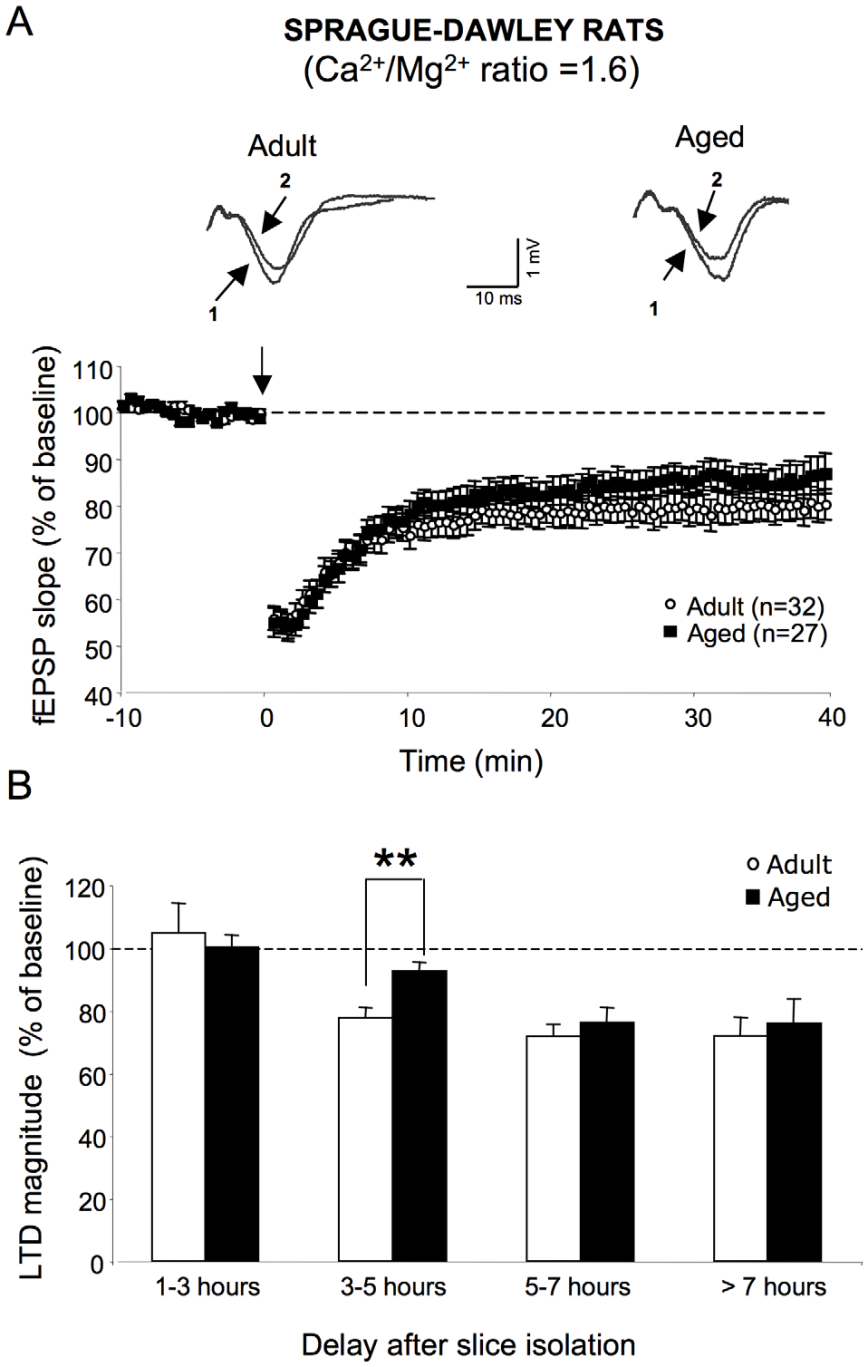
LFS-induced LTD is differentially expressed in slices from adult and aged Sprague-Dawley rats depending on the rest period. **A, top**. Superimposed sample traces of evoked fEPSPs induced by the electrical stimulation of glutamatergic afferents in the *stratum radiatum* of an adult (left) and aged Sprague-Dawley rat (right) before (1) and 40 min after LFS induction (2). **A, bottom**. Averaged LTD expressed as percent change in the slope of fEPSP vs. time, recorded in aCSF with a Ca^2+^/Mg^2+^ ratio of >1.5 in slices from adult (n = 32) and aged (n = 27) animals. Note that LTD is comparable in the two groups of animals. **B**. Comparison of LTD magnitude averaged from the last 10 min of recordings in adult and aged Sprague-Dawley rats as a function of the interval between slice isolation and LFS delivery (** *p*<0.01). Note an age-related decrease in LTD expression in slices that have rested for 3 to 5 h, which is not observed at longer delays.

When applied to slices from adult Sprague-Dawley rats (29 slices/18 animals) in aCSF with a Ca^2+^/Mg^2+^ ratio of 1.6, LFS produced a significant depression of fEPSPs that persisted until the end of the recording [F_1,57_ = 39.6, *p*<0.0001] (Figure 1A). In aged rats (27 slices, 17 animals), LFS also induced LTD [F_1,44_ = 15.7, *p*<0.001], which was not significantly different from the depression exhibited by young animals [F_1,49_ = 2.1, *p* = 0.1] (Figure 1A). However, studying susceptibility to LTD as a function of the delay after slice isolation revealed clear differences between the two groups of animals (Figure 1B). LFS delivered to slices shortly after slicing (1–3 h) did not induce LTD either in slices from adult rats (n = 5) or from aged ones (n = 5). In slices that were allowed to rest for 3 to 5 hours, LTD was induced by LFS in adult rats (77.7±3.1% of baseline value) [F_1,8_ = 0.2, *p*<0.0001, n = 14] but not in old animals (92.6±2.5% of baseline value) [F_1,8_ = 4.4, *p = *0.9, n = 8]. As a consequence, the magnitude of the depression was significantly weaker in slices from aged than from adult ones [F_1,17_ = 7.9, *p*<0.01] (Figure 1B). Longer rest periods after slicing (5 to 7 h) did not significantly increase LTD magnitude in adult rats (71.7±3.8% of baseline value) [F_1,20_ = 1.4, *p = *0.3, n = 8]. In contrast, the extent of LTD induced in slices from aged animals (76.1±4.7% of baseline value) was significantly enhanced compared to the weak depression generated at shorter delays [F_1,9_ = 3.9, *p*<0.05, n = 8] (Figure 1B). Consequently, the level of LTD expression was not significantly different in adult and aged rats at these delays between isolation and LFS induction [F_1,12_ = 1.1, *p = *0.3] (Figure 1B). At longer recovery times (>7 h), the magnitude of LTD was stabilized both in adult (71.9±5.9% of baseline value, n = 4) and aged (75.9±7.6% of baseline value n = 6) animals (Figure 1B).

### Effects of the NMDA-R antagonist d-APV

**Figure 2:**
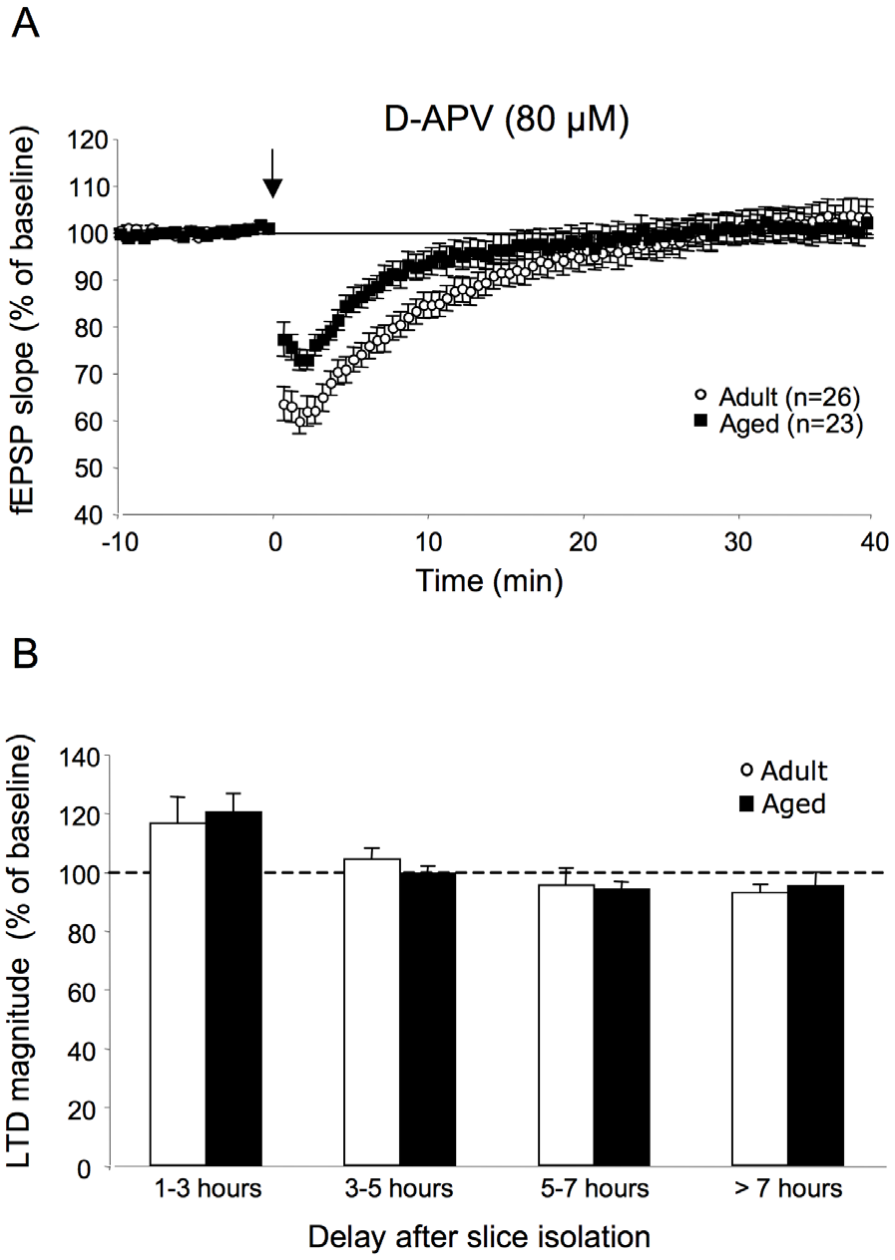
LFS-induced LTD is only mediated by NMDA-R activation in adult and aged rats. **A.** Averaged LTD expressed as percent change in the slope of fEPSP vs. time, recorded in slices from adult (n = 26) and aged (n = 23) Sprague-Dawley rats in the presence of the NMDA-R antagonist d-APV (80 µM). Note that d-APV prevents LTD expression in both groups of animals. **B.** Comparison of LTD expression as a function of the interval between slice isolation and LFS delivery in the two groups of animals in the presence of d-APV.

I then asked whether LTD induced by LFS depended solely on NMDA-R activation or whether additional sources could contribute to it as a function of recovery times of the slices. When all recordings were pooled (Figure 2A), LFS delivered in the presence of d-APV (80 µM) still initially depressed fEPSPs in adult rats (26 slices/11 animals) but responses rapidly returned to basal values, indicating that LTD expression was closely linked to NMDA-R activation. In aged rats (23 slices/8 animals), LTD was also completely antagonized by d-APV (Figure 2A). As illustrated in Figure 2B, no statistically relevant depression was induced by LFS in slices from either adult or aged rats treated with d-APV, regardless of the delay between slice isolation and LFS induction. These results indicate that under our experimental conditions, only NMDA-R was concerned by the LFS protocol. However, it is worth noting that unexpected and significant potentiations were induced in slices from adult and aged rats treated with d-APV and recorded 1–3 h after isolation (116. 2±9% of baseline value and 120.2±6.3% respectively) suggesting the activation NMDA-R-independent mechanisms under these conditions (Figure 2B). On the other hand, the fact that LTD expression was abolished by d-APV after long periods of rest in both groups of animals indicates that mechanisms that lead to the restoration of robust LTD in slices from aged rats at these intervals do not involve NMDA-R independent activation but may involve related to changes in the resting conditions of the slices.

### Effects of the NMDA-R co-agonist d-serine

The possible mechanisms underlying the age-related decrease in LTD expression 3 to 5 h after the slice isolation were then addressed using the NMDA-R co-agonist d-serine at saturating doses (100 µM). This procedure has been reported to reverse the deficit in theta-burst stimulation-induced LTP in aged Sprague-Dawley [29] or Wistar rats [30].

**Figure 3:**
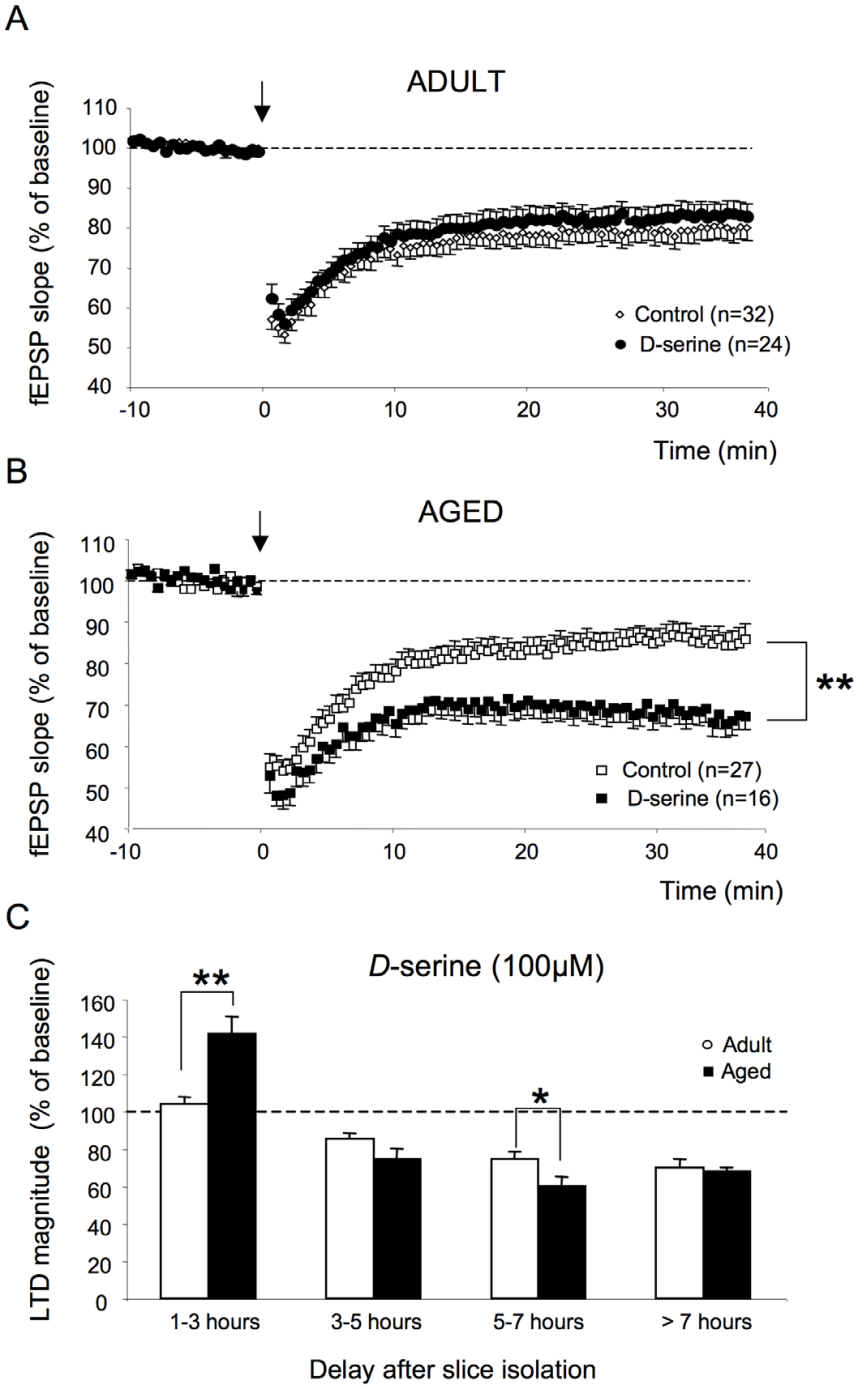
The NMDA-R co-agonist d-serine alters LTD expression in aged but not in adult rats. **A.** Averaged LTD expressed as percent change in the slope of fEPSP vs. time, recorded in adult Sprague-Dawley rats in control medium (n = 32) and in the presence of d-serine (n = 24). **B.** Same as in **A** but with recordings carried out in aged animals (control: n = 27; d-serine: n = 16). Note the significant increase in LTD magnitude in the presence of the NMDA-R co-agonist in aged rats but not in adults. (** *p*<0.01). **C.** Comparison of LTD expression as a function of the interval between slice isolation and LFS delivery in the two groups of animals in the presence of d-serine (** *p*<0.01, * *p*<0.05). Note the higher LTD induced in aged rats at the 5–7 h interval.

When all recordings were pooled (except for the 1–3 h group, see below), the magnitude of the depression was not affected by d-serine in adult rats (24 slices/13 animals) [F_1,43_ = 0.36, *p = *0.6] whereas it was significantly facilitated in aged animals (16 slices/11 animals) [F_1,41_ = 14.5, *p*<0.01] (Figure 3A and 3B). Consequently, under these conditions of saturation of NMDA-R glycine binding sites, the magnitude of LTD was significantly higher in slices from aged animals than in adult ones [F_1,33_ = 5.62, *p*<0.05].

There were also striking differences between adult and aged Sprague-Dawley rats with respect to the effects of d-serine on LTD expression as a function of the delay after slice preparation (Figure 3C). After 1 to 3 h of rest, no effect of the NMDA-R co-agonist was detected in the former group [F_1,8_ = 0.01, n = 5, *p = *0.9] whereas a significant potentiation (141.4±9.1% of baseline value) was induced in the latter [F_1,9_ = 21.5, *p*<0.01, n = 6]. For periods from 3 to 5 h, LTD expression in adult animals was not changed by d-serine [F_1,19_ = 2.24, *p = *0.2, n = 7] whereas the level of depression was significantly increased in old animals [F_1,13_ = 9.83, *p*<0.01, n = 7]. As a consequence, the age-related impairment that is normally observed under control conditions at these delays following slice recovery was alleviated in the presence of the NMDA-R co-agonist (Figure 3C). At longer rest periods (5–7 h), LTD expression with or without d-serine was again comparable in adult rats [F_1,14_ = 0.28, *p = *0.6, n = 8] whereas LTD was still enhanced by the co-agonist in aged animals [F_1,12_ = 5.25, *p*<0.05, n = 6]. Interestingly, LTD expression in the presence of d-serine at this point appeared to be significantly higher in aged than in adult animals [F_1,12_ = 5.87, *p*<0.05] (Figure 3C). Finally, there was no significant effect of d-serine on LTD expression in either adult [F_1,17_ = 0.29, *p = *0.8, n = 4] or aged [F_1,6_ = 0.41, *p = *0.5, n = 3] rats at slice recovery delays of more than 7 h (Figure 3C).

### Age-related susceptibility to LTD in Wistar rats

In order to determine whether the dependence of susceptibility to LTD on the delay between slicing and LFS induction is specific to Sprague-Dawley rats, a similar investigation was carried out in adult (n = 8) and aged (n = 6) Wistar rats.

**Figure 4:**
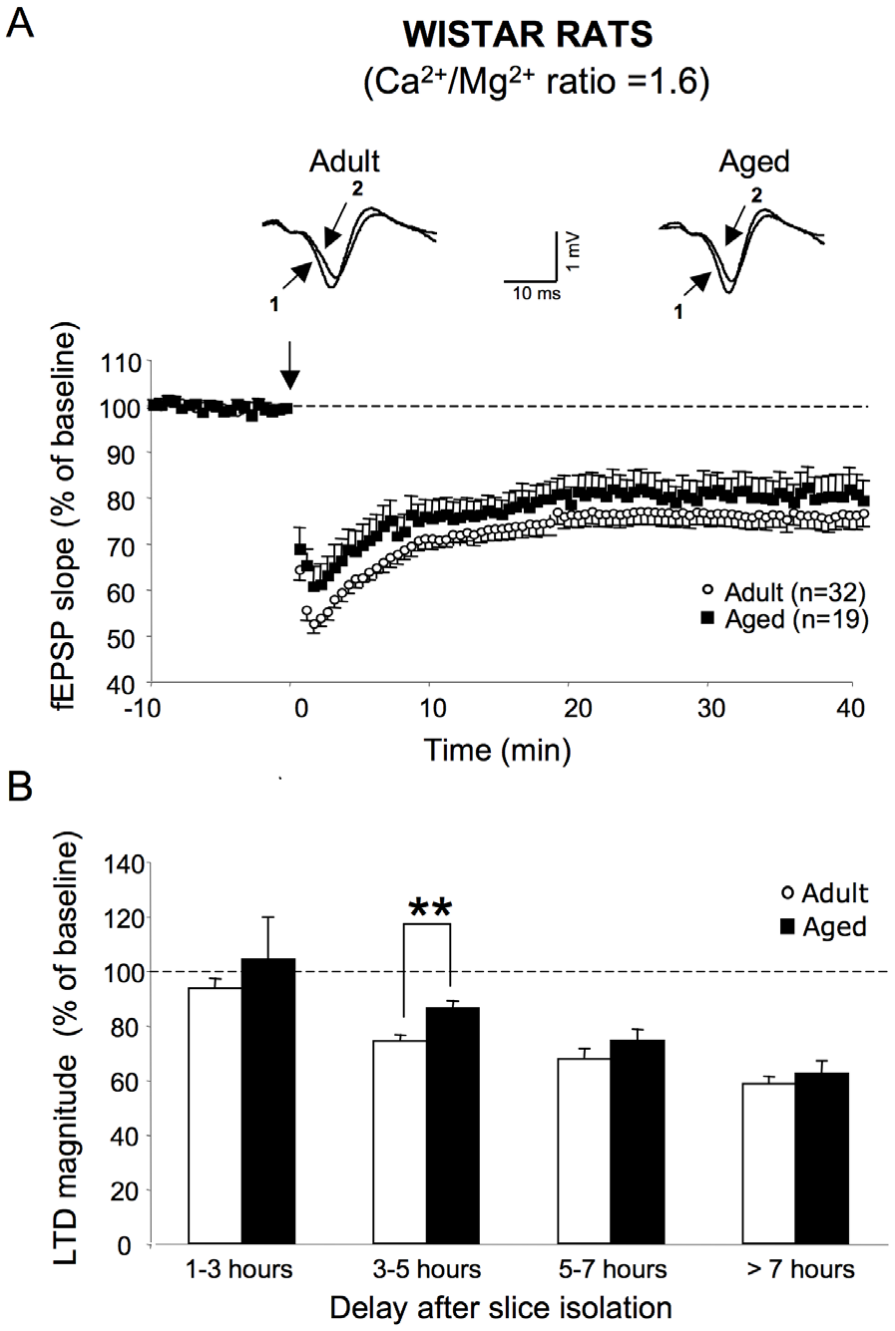
LFS-induced LTD is differentially expressed in slices from adult and aged Wistar rats depending on rest period. **A, top**. Superimposed sample traces of evoked fEPSPs induced by the electrical stimulation of glutamatergic afferents in the *stratum radiatum* of an adult (left) and aged Wistar rat (right) before (1) and 40 min after LFS induction (2). **A, bottom**. Averaged LTD expressed as percent change in the slope of fEPSP vs. time, recorded in aCSF with a Ca^2+^/Mg^2+^ ratio of >1.5 in slices from adult (n = 32) and aged (n = 19) animals. **B**. Comparison of LTD magnitude in adult and aged Wistar rats as a function of the interval between slice isolation and LFS delivery (** *p*<0.01). Note the same time-course as in Figure 1B for Sprague-Dawley rats.

When all recordings were pooled, LFS led to LTD in slices from both adult [F_1,62_ = 79.8, *p*<0.0001, n = 32] and aged rats [F_1,44_ = 18.2, *p*<0.001, n = 19]. Under these conditions, no age-related differences was observed [F_1,48_ = 2.3, *p = *0.1] (Figure 4A). Regarding LTD susceptibility as a function of the delay after slice isolation, no noticeable depression was induced in either group of animals for slices analyzed 1 to 3 h after the isolation procedure (Figure 4B). In slices that were allowed to rest for 3 to 5 h, LTD was expressed in adult (74.2±2.3% of baseline value) [F_1,14_ = 126.7, *p*<0.0001, n = 8] as well as in old rats (86.26±2.7% of baseline value) [F_1108_ = 29, *p*<0.001, n = 6). However, the level of depression was significantly greater in slices from adults than in those of aged ones [F_1,12_ = 11.5, *p*<0.01] (Figure 4B). At rest periods of 5 to 7 h, LTD magnitude was not changed in adult animals, as compared to earlier intervals (67.6±3.8% of baseline value) [F_1,15_ = 2.1, *p = *0.2, n = 8]. In contrast, the level of LTD was significantly enhanced in aged animals (74.5±3.9% of baseline value) [F_1,10_ = 5.9, *p*<0.05, n = 6] (Figure 4B). Consequently, LTD expression was comparable in adult and aged rats for these delays between slice isolation and LFS induction [F_1,12_ = 1.2, *p = *0.3] (Figure 4B). At delays of more than 7 h, the level of LTD in adult (58.5±2.6% of baseline value, n = 7) and aged (62.4±4.6% of baseline value, n = 4) animals was stabilized, and no age-related difference was observed (Figure 4B). Because these results mostly reproduced those observed in Sprague-Dawley rats, they appear to reflect a general property of hippocampal tissues to the effects of aging on LTD susceptibility.

### Effects of altering the Ca^2+^/Mg^2+^ ratio on susceptibility to LTD induction

Several lines of evidence in the literature indicate that LTD expression induced by a 1 Hz-conditioning stimulation, such as that used in the present study, is closely dependent on the Ca^2+^/Mg^2+^ ratio (see [31] for a review). It has been reported that LFS fails to induce LTD in young animals when this ratio is closed to 1, whereas significant depression may be induced under the same conditions in aged rats [12], [14], [15]. In this section, I therefore investigated age-related changes in LTD induction as a function of the delay between slice isolation and LFS administration in the presence of varying Ca^2+^/Mg^2+^ ratio.

**Figure 5:**
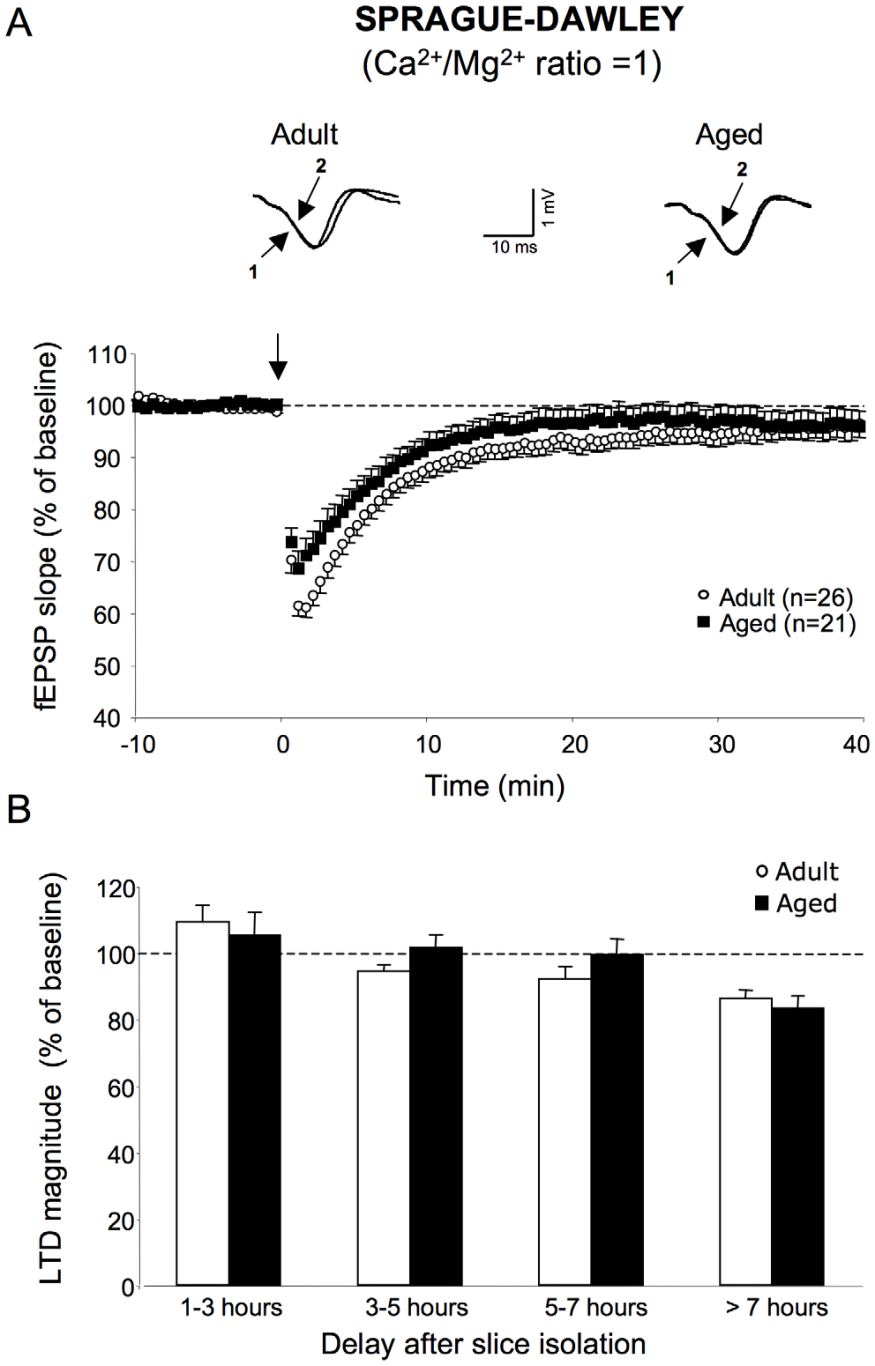
Effects of lowering the Ca^2+^/Mg^2+^ ratio to 1 on LTD expression in adult and aged Sprague-Dawley rats. **A, top.** Superimposed sample traces of evoked fEPSPs induced by the electrical stimulation of glutamatergic afferents in the *stratum radiatum* of an adult (left) and aged Sprague-Dawley rat (right) before (1) and 40 min after LFS induction (2). **A, bottom.** Averaged LTD expressed as percent change in the slope of fEPSP vs. time, recorded in slices from adult (n = 26) and aged (n = 21) animals. Note the absence of significant LTD in the two groups of animals. **B.** Comparison of LTD magnitude in slices as a function of the rest period (* *p*<0.05). Note that significant LTD is induced only at the longest delays after slicing, and that it is comparable in magnitude in adult and aged rats.

As illustrated in Figure 5A, no LTD was induced in adult (95.1±2.3% of baseline value, n = 26) [F_1,48_ = 3.53, *p = *0.07] or aged (96.6±2.8% of baseline value, n = 21) [F_1,48_ = 1.7, *p = *0.2] Sprague-Dawley rats in aCSF with a Ca^2+^/Mg^2+^ ratio of 1 when all recordings were pooled. This absence of depressive effect of LFS was also observed for periods of rest of up to 7 h (Figure 5B). At longer experimental times, LTD occurred in slices from adult animals (86.2±2.5% of baseline value, n = 6) [F_1,10_ = 30.8, *p*<0.01] as well as from aged ones (83.3±3.7% of baseline value, n = 5) [F_1,10_ = 22.1, *p*<0.01] with no statistically significant difference between the two groups [F_1,10_ = 0.3, *p = *0.6] (Figure 5B).

## Discussion

The present study provides evidence that the magnitude of LFS-induced LTD mediated by the NMDA subtype of glutamate receptors is related to the interval between slice isolation and LFS induction, and that this correlation exists regardless of the Ca^2+^/Mg^2+^ ratio of the perfusion medium. In addition, it shows that differences in susceptibility to LTD between adult and aged Sprague-Dawley or Wistar rats are present or absent depending on the time for which slices are allowed to rest before recording, which may partly explain the discrepancy seen in the literature concerning susceptibility to LTD and aging.

With respect to the expression of long-lasting depression in an CSF with a Ca^2+^/Mg^2+^ ratio of 1, no LTD occurs in adult animals, as repeatedly reported (see [32] for a review). However, weak but noticeable LTD can be induced by LFS, but only in slices that are allowed to rest for more than 7 h. A similar profile of LTD expression is found in slices from aged animals, a finding that is different from several reports showing an age-related increase in LTD expression under the same Ca^2+^/Mg^2+^ ratio conditions [11], [12], [13], [14], [15], [16], [17]. Nevertheless, the dependence of LTD expression on the period for which slices are allowed to rest, described in the present study, could account for this discrepancy. Indeed, it may be hypothesized that the age-related increase in LTD magnitude reported by others is simply the result of differences in the delay between the slice preparation and recording in the two groups of animals. In adult rats, in which it is possible to rapidly isolate slices and in which fEPSPs are easily recorded, LFS could be administrated after a relatively short rest periods (<7 h), when no significant LTD is exhibited (see Figure 5B). In contrast, slices are usually less rapidly isolated in aged rats and electrophysiological recordings are often delayed in these animals due to difficulties in obtaining stable fEPSPs. Consequently, LFS could be induced in slices subjected to longer periods of rest (>7 h) than in those from than in adult animals, at a time when weak but significant LTD takes place.

In aCSF with a Ca^2+^/Mg^2+^ ratio of >1.5, LFS induces NMDA-R-dependent LTD in adult animals, as previously reported [18], [20], [33]. However, a minimal rest period of 3 h is required after slicing for the expression of a significant long-lasting depression. The magnitude of LTD then increases concomitantly with the rest period up to 5 h, and stabilizes over longer intervals. In the presence of this Ca^2+^/Mg^2+^ ratio, slices from aged rats behave differently. Even after a 5 h period of rest, no significant LTD is seen in these animals, due to the impaired activation of NMDA-R glycine binding sites. Indeed, age-related deficits in LTD are prevented when LFS is delivered in the presence of saturating concentrations of the endogenous NMDA-R co-agonist d-serine, similar to the effect of the related compound, d-cycloserine [18]. A reduction in the activation of NMDA-R glycine binding site by d-serine has recently been characterized in aged Sprague-Dawley [28], [29] and Wistar rats [30] accounting for the impaired theta-burst-induced LTP displayed by these animals.

Interestingly, when delivered to slices from aged animals allowed to rest more than 5 h, potent LTD occurs, comparable to the depression recorded in adult animals, indicating that compensatory mechanisms have taken place. At these delays separating slice isolation from LFS administration, it is interesting to note that LTD is significantly higher in aged than in adult rats if impaired NMDA-R activation is over come by saturating doses of d-serine (see Figure 3C). NMDA-R-independent mechanisms, such as the activation of L-type Ca^2+^ channels [13] and intracellular Ca^2+^ stores [12], have been reported to contribute to LTD expression in aged rats. However, a significant contribution of these pathways, or other such as the activation of metabotropic glutamate receptors [34], [35] to the compensatory mechanisms is unlikely since d-APV completely antagonizes LTD expression regardless of the recovery period of the slices. On the other hand, this result rather suggests that changes in resting conditions must instead be considered.

At CA3-CA1 synapses, a prolonged but moderate increase in postsynaptic intracellular free calcium ([Ca^2+^]i) is a prerequisite for the induction of LTD [23], [24], [25], [26]. One can therefore intuitively postulate that changes in basal [Ca^2+^]i will affect LTD expression by facilitating or preventing its induction as the basal [Ca^2+^]i level increases or decreases. Interestingly, it has been reported that in cerebellar granule neurons in brain slices, basal [Ca^2+^]i levels increase as a function of the time the slices are allowed to rest *in vitro*, and that after a delay of 3–4 h, the magnitude of this effect is significantly greater in slices from aged animals than in those from adult ones (see [27]). This weaker capacity of aged neurons to maintain a stable resting Ca^2+^ concentration has been demonstrated in slices as well as in cell culture preparations and has been primarily attributed to the inability of mitochondria to function as normal Ca^2+^ stores [36], [37]. Although this possibility remains to be definitively demonstrated, it is thus conceivable that similar changes in basal [Ca^2+^]i induced by slice isolation occur in hippocampal neurons, accounting for the time-dependence of LTD induction as well as for the weaker initial LTD in aged rats that is not observed at longer periods of slice recovery. At very short rest period (1–3 h), basal [Ca^2+^]i levels in neurons could be too low to allow the NMDA-R-dependent Ca^2+^ entry induced by LFS to reach the threshold required for LTD expression. After 3 to 5 h of rest, as the resting [Ca^2+^]i increases, this threshold may be reached in adult rats but not in aged ones, because of impaired NMDA-R activation. At longer times after slicing (5 to 7 h), the decrease in Ca^2+^ entry through the NMDA-R could be compensated by the greater increase in basal [Ca^2+^]i in aged neurons, alleviating the age-related impairment of LTD. This hypothesis could also explain why LTD is higher in aged animals than in adult ones at long recovery times, when the impairment in NMDA-R activation is prevented by d-serine (see Figure 3C).

Although attractive, these hypotheses would be difficult to prove because the manipulation of the Ca^2+^ environment of neurons would affect not only basal [Ca^2+^]i but also the Ca^2+^ dynamics induced by LFS. Alternatively, the age-related inability of mitochondria to function as normal Ca^2+^ stores could be reproduced in adult animals using the mitochondrial protonophore carbonyl cyanide p-(trifluoro-methoxy) phenylhydrazone (FCCP) [38], [39] in order to determine its effects on LTD expression. However, FCCP not only modifies basal [Ca^2+^]i in neurons but also significantly alters the amplitude of glutamate-induced Ca^2+^ transients [40], thus interfering with the process of LTD.

Although the use of slices rather than other preparations such as synaptosomes or dissociated neurons to investigate the physiology of aging has been the subject of debate [41], [42], it is obvious that slices are an adequate preparation in which to simultaneously control [Ca^2+^]i dynamics and electrophysiological responses. However, the present study points to an experimental “window of opportunity” for studying the effects of aging in hippocampal slices, at least those dependent on changes in basal [Ca^2+^]i. The crucial point is to know whether basal Ca^2+^ is really enhanced in aged neurons *in vivo*? If the answer is yes, then age-related LTD susceptibility must be investigated in slices that have rested for long periods and that display higher values of [Ca^2+^]i [27]. From the present study, it appears under these conditions that susceptibility to LTD is not modified in aged rats. Alternatively, if the answer is no, i.e. if basal [Ca^2+^]i is not enhanced in aged neurons *in vivo*, LTD *in vitro* needs to be studied shortly after slice isolation, when basal [Ca^2+^]i levels are similar in adult and aged neurons [27]. Under these conditions, we have shown that susceptibility to LTD is impaired in aging. Unfortunately, no data are yet available *in vivo* regarding the expression of LTD in the hippocampus of aged animals, that could favorite one hypothesis from another. It is worth noting that *in vivo* recordings in young animals also lead to discrepancy since some studies have indicated failure to induce LTD [43], [44] whereas others have shown successful induction in anesthetized or freely moving rats [45], [46]. Variations in rat strains and also in the behavioral state of the animal have been proposed to underlie the discrepancy [47] but changes in basal [Ca^2+^]i in neurons between studies could also be possibly be involved.

Although this study focuses on LTD expression, it is obvious that the length of time for which slices are allowed to rest may impact other Ca^2+^-dependent functional events analyzed in the slice preparations such as post-burst afterhyperpolarization potentials (AHPs). Indeed, it is worth noting that an age-related increase in AHPs has been reported in some studies (reviewed in [31]) possibly reflecting the result of intracellular recordings carried out at different intervals after slicing. On the other hand, time-dependent changes in basal [Ca^2+^]i may represent only one factor that interferes with the expression of synaptic plasticity in slice preparations. Indeed, a time-dependent loss of AMPA receptor subtypes GluR1 and GluR3 has been reported in slices from young rats suggesting that progressive changes may occur in the composition of major glutamate receptors [48]. This study also shows a time-dependent induction of mRNAs for the transcription factors *c-fos* and *zif268* and the neurotrophin brain-derived neurotrophic factor, which are all involved in the expression of synaptic plasticity. These modifications may therefore also contribute to the differences in LTD expression in slices from adult and aged animals as a function of time after slicing, although to what extent they occur in aged tissues has yet to be determined.
